# Identification of diagnostic candidate genes in COVID‐19 patients with sepsis

**DOI:** 10.1002/iid3.70033

**Published:** 2024-10-08

**Authors:** Jiuang Li, Shiqian Pu, Lei Shu, Mingjun Guo, Zhihui He

**Affiliations:** ^1^ Department of Critical Care Medicine The Third Xiangya Hospital, Central South University Changsha Hunan China

**Keywords:** bioinformatics, Coronavirus Disease 2019, diagnosis, immune infiltration, machine learning, sepsis

## Abstract

**Purpose:**

Coronavirus Disease 2019 (COVID‐19) and sepsis are closely related. This study aims to identify pivotal diagnostic candidate genes in COVID‐19 patients with sepsis.

**Patients and Methods:**

We obtained a COVID‐19 data set and a sepsis data set from the Gene Expression Omnibus (GEO) database. Identification of differentially expressed genes (DEGs) and module genes using the Linear Models for Microarray Data (LIMMA) and weighted gene co‐expression network analysis (WGCNA), functional enrichment analysis, protein–protein interaction (PPI) network construction, and machine learning algorithms (least absolute shrinkage and selection operator (LASSO) regression and Random Forest (RF)) were used to identify candidate hub genes for the diagnosis of COVID‐19 patients with sepsis. Receiver operating characteristic (ROC) curves were developed to assess the diagnostic value. Finally, the data set GSE28750 was used to verify the core genes and analyze the immune infiltration.

**Results:**

The COVID‐19 data set contained 3,438 DEGs， and 595 common genes were screened in sepsis. sepsis DEGs were mainly enriched in immune regulation. The intersection of DEGs for COVID‐19 and core genes for sepsis was 329, which were also mainly enriched in the immune system. After developing the PPI network, 17 node genes were filtered and thirteen candidate hub genes were selected for diagnostic value evaluation using machine learning. All thirteen candidate hub genes have diagnostic value, and 8 genes with an Area Under the Curve (AUC) greater than 0.9 were selected as diagnostic genes.

**Conclusion:**

Five core genes (CD3D, IL2RB, KLRC, CD5, and HLA‐DQA1) associated with immune infiltration were identified to evaluate their diagnostic utility COVID‐19 patients with sepsis. This finding contributes to the identification of potential peripheral blood diagnostic candidate genes for COVID‐19 patients with sepsis.

## Graphical abstract

A systematic identification of five immune‐related diagnostic hub genes (CD3D, CD5, HLA‐DQA1, IL2RB, and KLRC1) was conducted in this study. This research is anticipated to establish a foundation for clinical diagnosis and enhance the prognosis of sepsis patients in COVID‐19.

## 1 INTRODUCTION

Sepsis is a severe and potentially fatal clinical syndrome and the leading cause of infection‐associated death. Based on data from seven high‐income countries, it is estimated that 31.5 million cases of sepsis occur globally each year, with a mortality rate of 17% for hospitalized sepsis and 26% for severe sepsis.^1^ The COVID‐19 pandemic has caused a sudden and significant increase in hospital admissions for pneumonia worldwide with more than 100 million confirmed COVID‐19 cases and more than 2 million associated deaths counted worldwide by the end of January 2021.^2^ According to a large body of clinical data, the leading causes of death in COVID‐19 are respiratory failure and sepsis. In fact, sepsis has been observed in almost all COVID‐19 deaths.^3^, ^4^, ^5^ It is well known that the prognosis of COVID‐19 is poor after it has progressed to sepsis, so it is important to find a sensitive and specific diagnostic tool for the early detection of sepsis in COVID‐19, which will help to limit internal tissue and organ damage.^6^, ^7^

Patients with severe COVID‐19 infections, as previously reported for sepsis, exhibit excessive inflammation and cytokine storms.^6^, ^8^ Immune inflammation serves as a crucial link between COVID‐19 and sepsis.^9^, ^10^ Proteomics and sequencing tools offer the potential to identify novel biomarkers and their role in various diseases.^11^ As machine learning matures in bioinformatics applications, it can unravel underlying mechanisms, potential biomarkers, and therapeutic targets for a multitude of diseases.^12^, ^13^, ^14^

Limited research exists on identifying diagnostic candidates and machine learning for COVID‐19 patients with sepsis.^14^ Prior efforts have primarily focused on identifying diagnostic biomarkers for sepsis or COVID‐19.^15^ However, it remains unclear which patients will develop sepsis upon detection of COVID‐19, making it imperative to investigate early peripheral diagnostic biomarkers in COVID‐19 patients for prompt intervention.^16^ This study may contribute to the identification of potential diagnostic markers for sepsis in COVID‐19 patients.^17^ The datasets of COVID‐19 and sepsis were retrieved from the GEO database.^18^ DEGs were identified using LIMMA, followed by the selection of module genes using WGCNA.^19^ Subsequently, functional enrichment analysis, PPI network construction, machine learning (RF and LASSO), ROC curve evaluation, validation of external datasets, and immune infiltration were performed to identify the key immune‐related diagnostic biomarkers of COVID‐19 with sepsis.^20^, ^21^

## 2 MATERIAL AND METHODS

### 2.1 Data source

**Figure 1:**
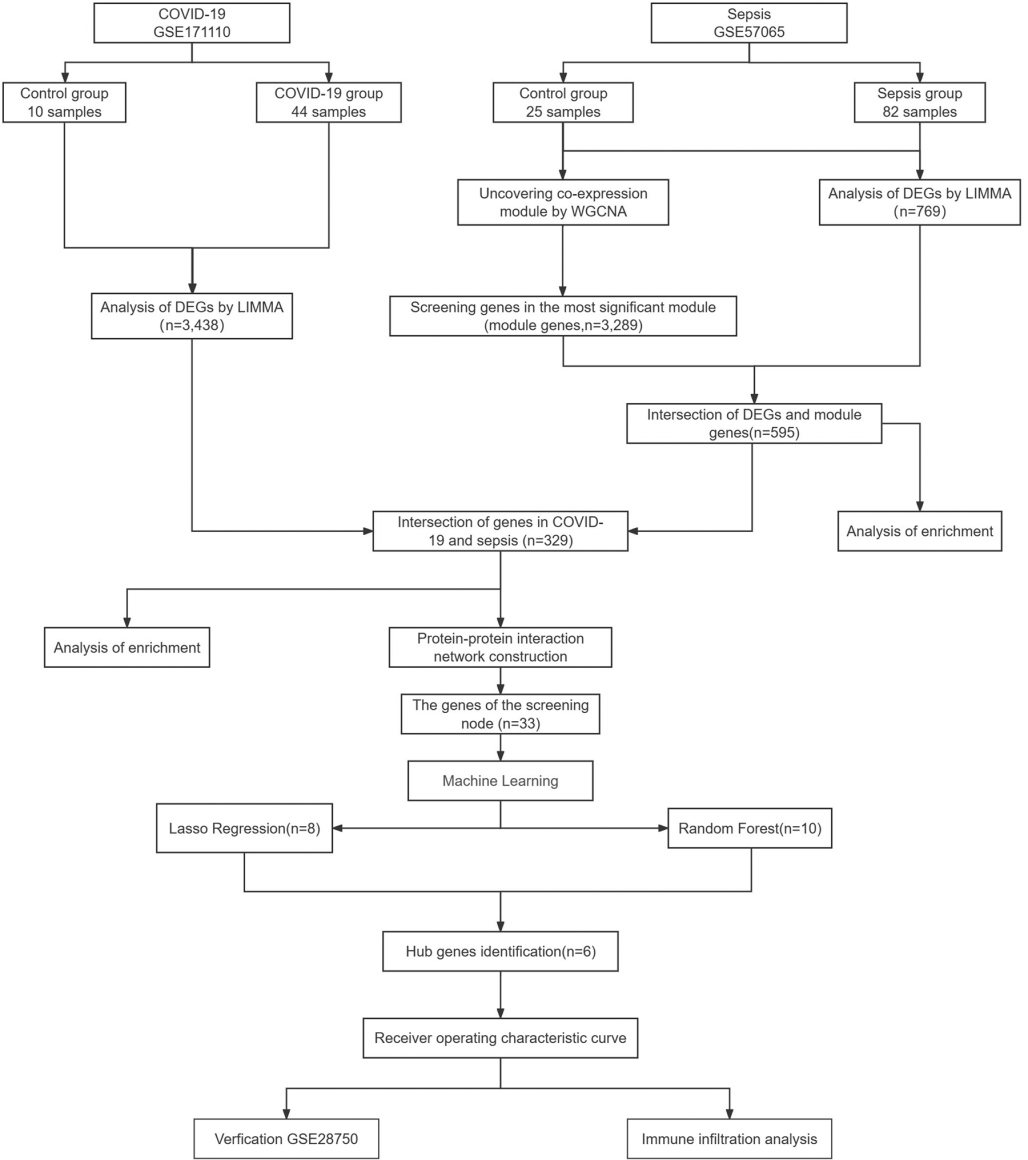
Flowchart of the study.

Figure 1 shows the study flowchart. We downloaded two datasets from the GEO (https://www.ncbi.nlm.nih.gov/geo/) database: the COVID‐19 data set GSE171110 and the sepsis data set GSE57065. Table 1 provides the information, including microarray platform, sample groups and numbers.

**Table 1:** Basic information on the GEO datasets used in the study. (GSE171110 includes severe COVID‐19 patients, and GSE57065 comprises 28 sepsis patients with three time points of blood collection (30 min, 24 h, and 48 h post septic shock), resulting in 82 samples).

GSE series	Type	Control	COVID‐19	Platform
GSE171110	mRNA	10	44	GPL16791
	Control	Sepsis		
GSE57065	mRNA	25	82	GPL570

### 2.2 Data processing and differentially expressed gene screening

We generate the representation matrix, eliminate rows containing more than 50% NA values, and columns with more than 50% NA values, and then employ the R package impute to fill in missing data via log2 transformation. Next, we set the criteria for identifying DEGs using the Limma package, which involves |log2 Fold change (FC)| > 1 and *p*‐value < .05.

### 2.3 Weighted gene co‐expression key gene and module selection

To identify potential genes associated with sepsis, we employed WGCNA to investigate gene associations in 82 peripheral blood samples from sepsis patients and 25 peripheral blood samples from healthy controls. Firstly, we determined the median absolute deviation (MAD) for each gene and eliminated 50% of the genes with the smallest MAD. Secondly, we filtered the DEGs expression matrix using the goodSamplesGenes function to exclude unqualified genes and samples, followed by constructing a scale‐free co‐expression network. Thirdly, we calculated adjacency using a “soft” threshold (β) derived from co‐expression similarity. Subsequently, we converted adjacency into a topological overlap matrix (TOM) and assessed gene ratio and dissimilarity. The fourth step involved detecting modules through hierarchical clustering and dynamic tree‐cutting functions. Genes exhibiting similar expression profiles were grouped into gene modules based on average linkage hierarchical clustering with TOM‐based dissimilarity metric, where a minimum gene group size of n = 300 was applied for the gene dendrogram. Fifthly, module eigengene dissimilarity was computed to select a cut line for module dendrogram and merge several modules for further investigation. Finally, the target genes were subsequently identified by intersecting the key genes identified through WGCNA screening with the DEGs.

### 2.4 Functional enrichment analysis

The R package clusterProfiler was employed for conducting GO analysis and KEGG analysis, with a significance threshold of *p* < .05 to determine statistically significant differences.^22^ The enrichment analysis results were then visualized using the Sangerbox platform (http://sangerbox.com/).

### 2.5 PPI network construction and gene screening

The interactions between protein‐coding genes were explored by constructing a PPI network using the String database(https://cn.string-db.org/), with a minimum interaction score threshold set at 0.400{Fang, 2023 #404}. The images obtained from String were further refined using Cytoscape software, and significant interacting genes were identified utilizing the MCODE plug‐in.

### 2.6 Machine learning

The LASSO regression technique was employed as a variable selection and regularization method to enhance the predictive accuracy and interpretability of statistical models, thereby facilitating the screening of candidate genes for sepsis diagnosis. Additionally, the RF method, which combines ensemble learning algorithms with machine learning techniques, was utilized due to its ability to predict continuous variables without being constrained by variational conditions or requiring significance testing.^23^ In this study, we performed LASSO regression analysis using the R package. Additionally, RF analysis was conducted using the publicly available platform Wekemo Bioincloud (https://bioincloud.tech/).^23^ The intersection of genes identified by both LASSO and RF approaches were considered as potential hub genes in sepsis diagnosis.

### 2.7 Receiver operating characteristic evaluation

We utilized the R package pROC (version 1.17.0.1) to establish the ROC for evaluating the diagnostic efficacy of candidate genes, and quantitatively measured its value by calculating the area under the curve (AUC) along with its 95% confidence interval (CI). The AUC greater than 0.9 was considered as the optimal diagnostic index.

### 2.8 Validation of hub gene expression

The GSE28750 data set, that contains 10 sepsis patients and 20 healthy control samples, was used to verify the expression levels of the key genes in this study. Wilcoxon test was used to compare the data between the two groups. A comparison between the two datasets was conducted using the Wilcoxon test. A *p* value < .05 was deemed significant.

### 2.9 Immune infiltration analysis

Immune cell infiltration analysis was conducted using the “Cibersort” R package, and Spearman correlation analysis was employed to determine the correlation between the five hub genes and immune cells, unveiling the interrelation between these genes and immune cells.

## 3 RESULTS

### 3.1 Identification of differentially expressed genes

**Figure 2:**
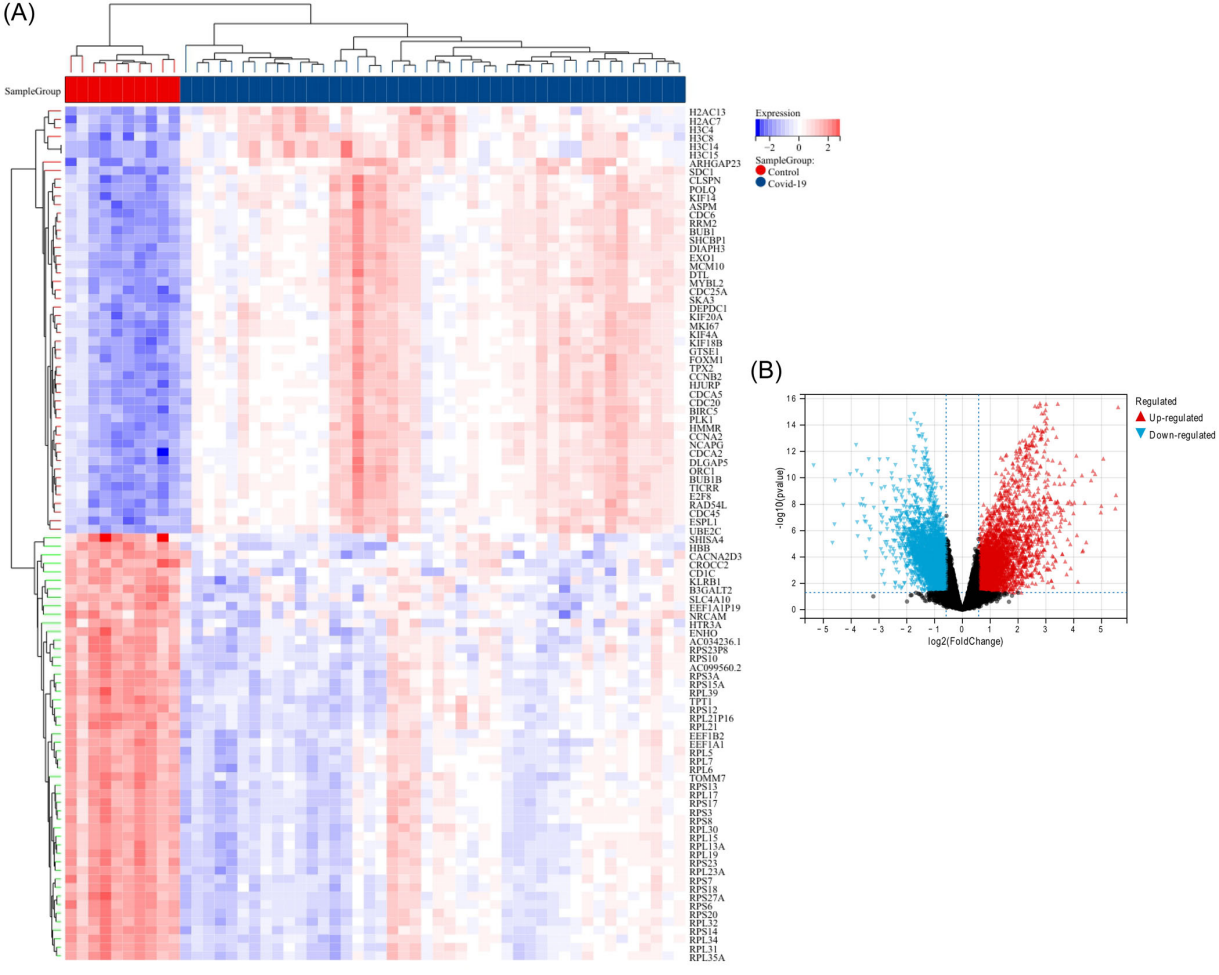
(A) Heatmap of DEGs in GSE171110. (B) Volcanic plot of DEGs in GSE171110.

**Figure 3:**
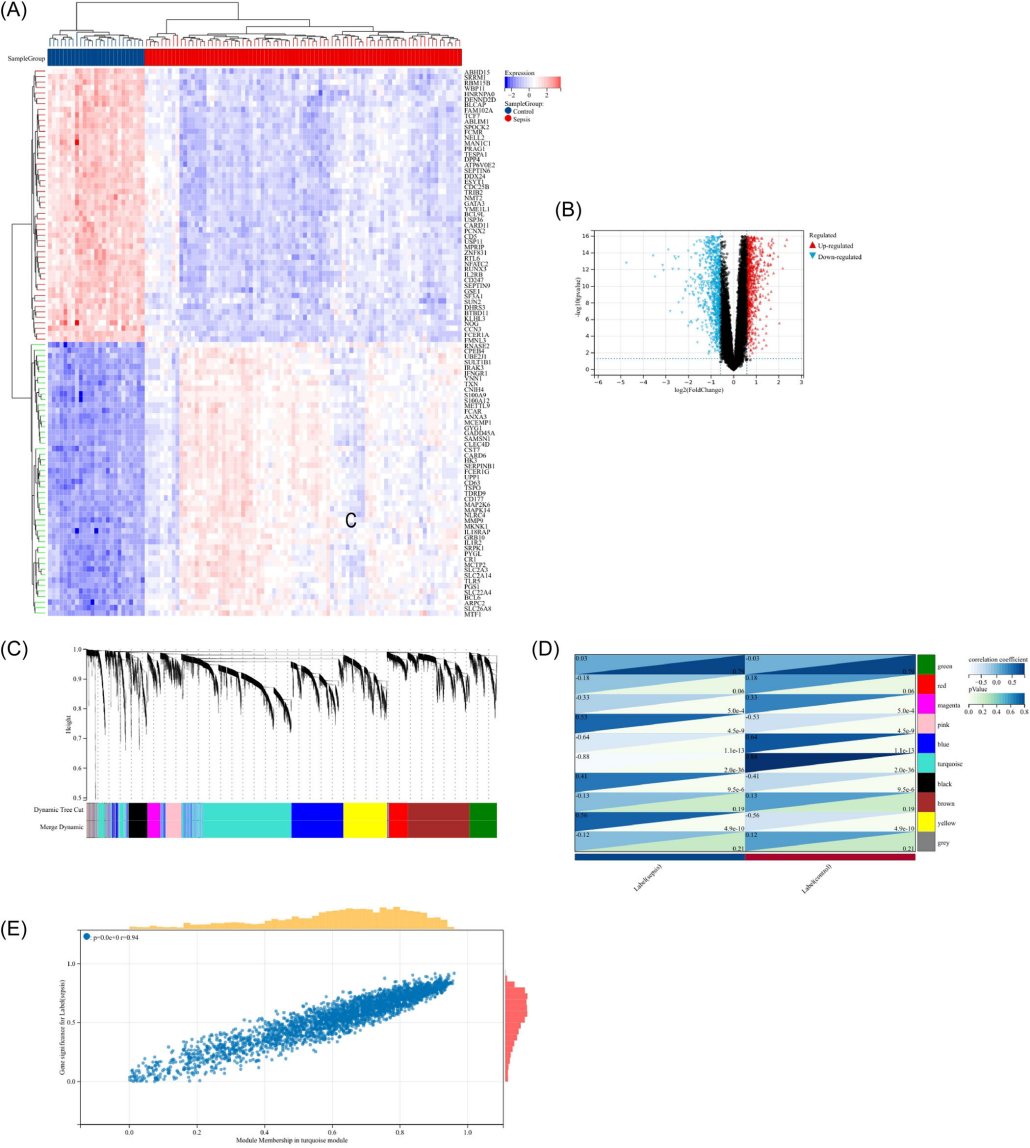
The DEGs in sepsis were identified using the Limma, while the module genes were identified through WGCNA. The heatmap (A) and volcano (B) presented the expression of DEGs in GSE57065.β = 6 is selected as the soft threshold with the combined analysis of scale independence and average connectivity. (C) The gene tree depicts distinct gene co‐expression modules, each represented by a unique color. (D) Heatmap of the association between modules and sepsis. Turquoise modules were significantly associated with sepsis. (E) The correlation plot depicts the relationship between module membership and gene significance of genes that are encompassed within the turquoise module.

A total of 3,438 DEGs were identified in the COVID‐19 data set using the Limma method, with 1,872 were upregulated and 1,566 downregulated DEGs. The DEGs from the COVID‐19 datasets were utilized to generate a heatmap and volcano map (Figure 2A, B). In the sepsis data set, we screened 769 DEGs (351 upregulated and 418 downregulated) (Figure 3A, B).

### 3.2 WGCNA and key module identification

The most correlated module in sepsis was identified using WGCNA. A “soft” threshold of β = 6 (scale‐free R2 = 0.88) was chosen based on scale independence and average connectivity. In Figure 3C with different colors to describe different gene expression of the tree. The correlation between sepsis and gene co‐expression modules is illustrated in Figure 3D, and the turquoise module (consisting of 3,289 genes) exhibiting the highest correlation with sepsis (correlation coefficient =−0.88, p = 2.0 * 10^−36^), thus considered as the central module for further analysis. Furthermore, a strong correlation(r = .94) between module membership and gene significance within the turquoise module for sepsis was calculated, indicating that the genes most significantly associated with sepsis were located within this particular module.

### 3.3 Analysis of the functional enrichment of sepsis

**Figure 4:**
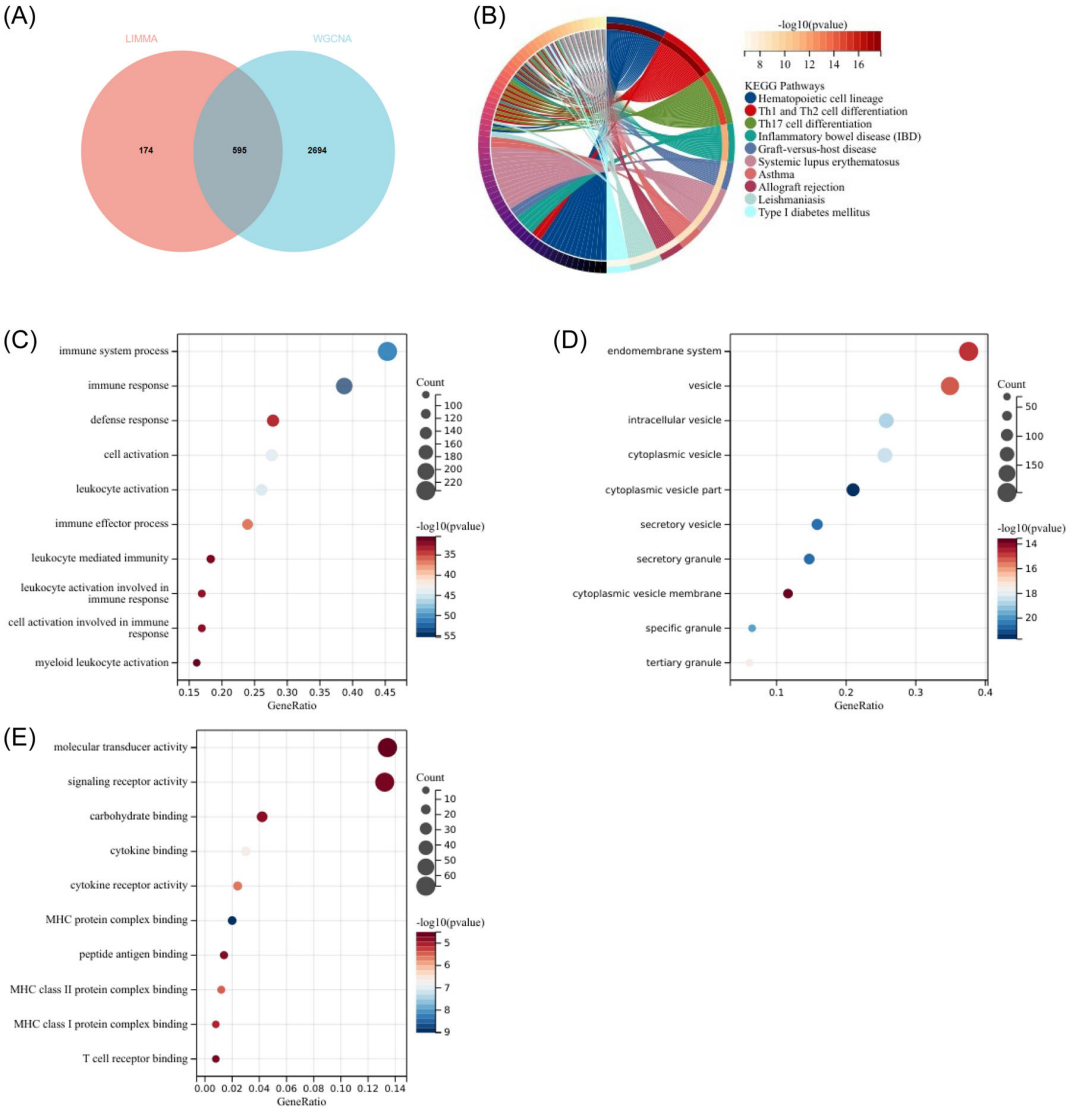
Enrichment analysis of the intersection of genes in sepsis. (A) A Venn diagram illustrating the overlap between DEGs and genes belonging to the turquoise module in WGCNA for sepsis. (B) KEGG pathway analysis of the intersection of genes. (C–E) GO analysis of the intersection of genes, including biological process (BP), cellular component (CC), and molecular function (MF), respectively.

To assess the relevance of the GSE57065 data set in representing sepsis, we conducted functional enrichment analysis by intersecting genes identified through LIMMA and WGCNA module genes. Through this intersection, a total of 595 common genes were identified from the overlap between 769 DEGs and 3,289 genes in the turquoise module (Figure 4A).

The KEGG analysis revealed that the common genes were predominantly enriched in the “Hematopoietic cell lineage” and “Th1 and Th2 cell differentiation” pathways (Figure 4B). GO analysis demonstrated that for BP, these genes were primarily enriched in “immune system process” and “immune response” (Figure 4C). In terms of CC, the genes were mainly enriched in the “endomembrane system” and “vesicle” (Figure 4D). Furthermore, for MF, the genes were mostly enriched in “molecular transducer activity” and “signaling receptor activity” (Figure 4E).

Enrichment analysis revealed that the genes associated with sepsis were predominantly linked to immune and inflammatory responses, indicating a strong correlation between these processes and the processes and the pathogenesis of sepsis. These findings establish a reliable foundation for subsequent analysis of COVID‐19.

### 3.4 Enrichment analysis of COVID‐19 patients with sepsis, construction of PPI network and identification of key genes

**Figure 5:**
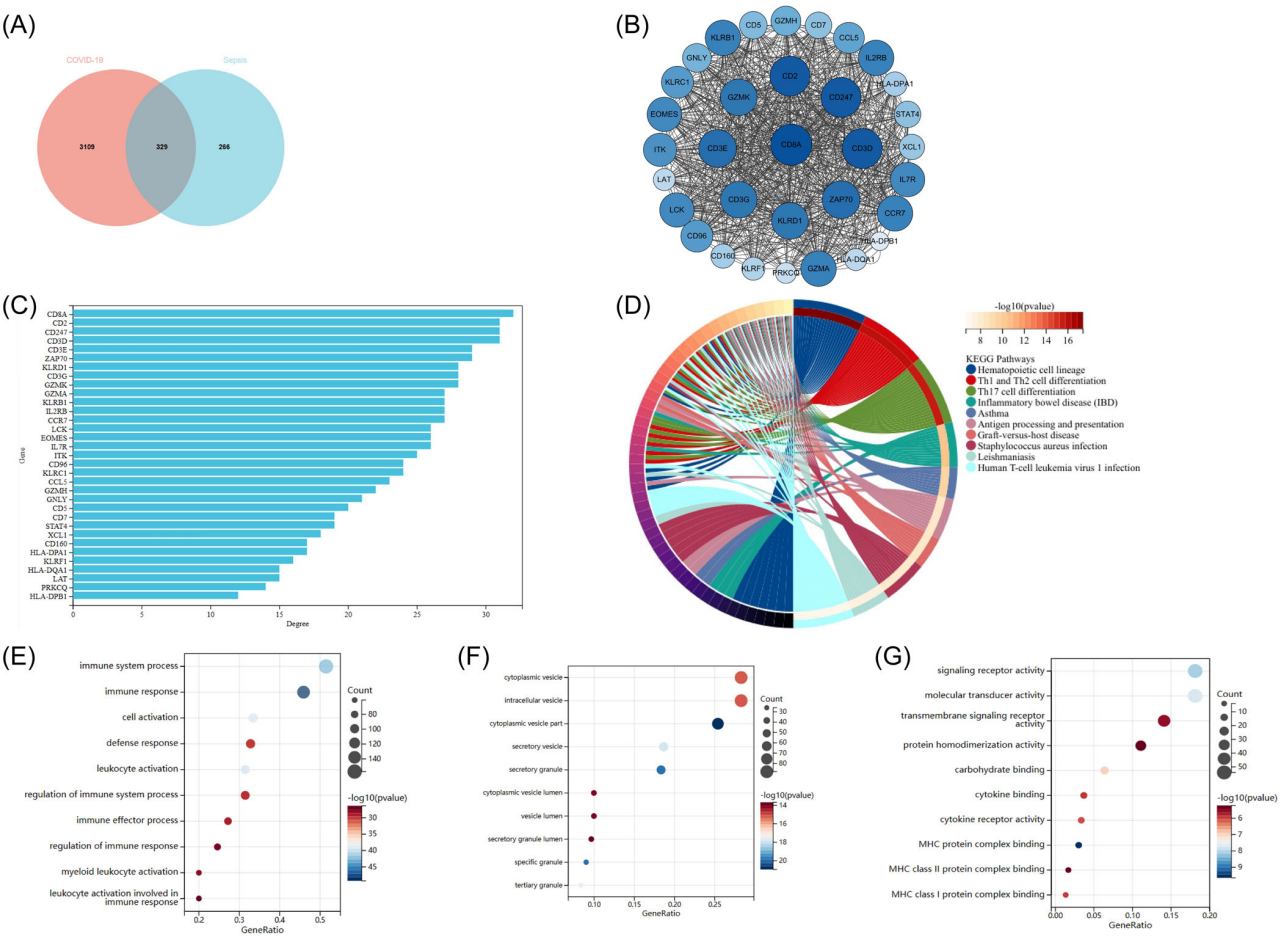
Enrichment analysis was performed to identify CGs between COVID‐19 and sepsis, followed by the identification of key node genes from the PPI network. (A) Venn diagram. (B) The most prominent modules in the PPI network are visualized using the MCODE plug‐in. (C) The column presents the gene nodes of 33 genes in PPI network. (D) KEGG analysis of 329 CGs. (E–G) GO analysis (BP, CC and MF).

To further investigate the potential relationship between sepsis‐associated key genes and COVID‐19, we identified 329 genes from the intersection of DEGs in COVID‐19 and sepsis candidate genes (CGs), as visualized by a Venn diagram (Figure 5A). The KEGG enrichment analysis revealed that these 329 genes were primarily enriched in pathways related to “Hematopoietic cell lineage,” “Th1 and Th2 cell differentiation,” and “Th17 cell differentiation”; all of which are closely associated with immune system function (Figure 5D). Furthermore, GO analysis demonstrated enrichment in BP such as “immune system process,” “immune response，” and “cell activation” (BP); CC including “cytoplasmic vesicle,” “intracellular vesicle,” and “cytoplasmic vesicle part” (CC); as well as MF like “signaling receptor activity,” “molecular transducer activity,” and “transmembrane signaling receptor activity” (MF) (Figure 5E‐G).

After establishing the intimate association of the screened genes with immunity, we constructed a PPI network to identify interacting node genes for subsequent machine learning‐based filtration. Figure 5B depicts the presence of 33 interacting genes in the PPI network, while Figure 5C hierarchically ranks these genes based on their node numbers.

### 3.5 Machine learning identification of candidate hub genes

**Figure 6:**
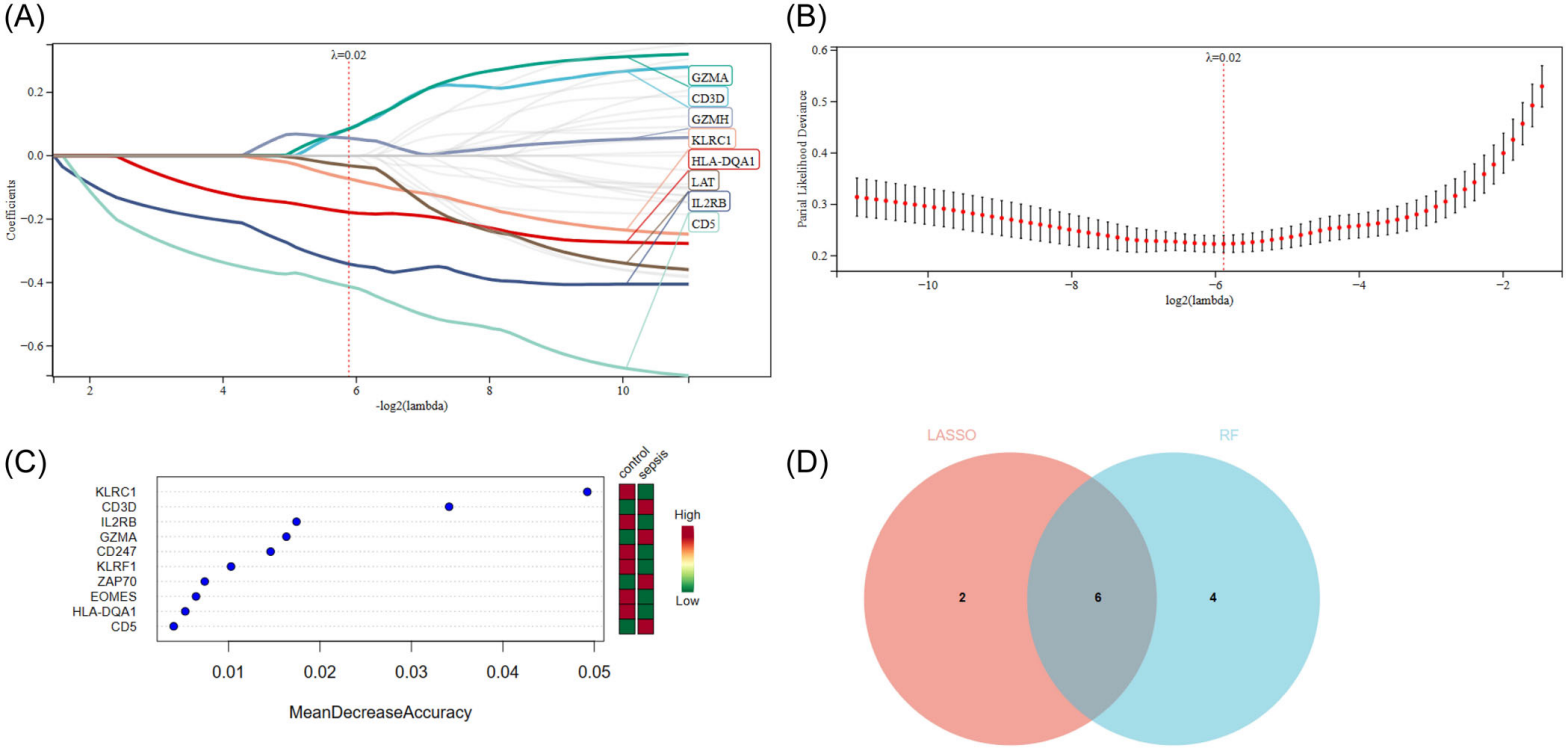
Machine learning LASSO screening was employed to identify candidate diagnostic genes for COVID‐19 with sepsis (A, B). The Random Forest algorithm was then utilized based on importance score ranking (C). Additionally, a Venn diagram demonstrated that the two algorithms collectively identified six potential diagnostic genes (D).

LASSO regression machine learning algorithm was used to screen 33 interacting genes in the PPI network for diagnostic value evaluation. Eight candidate genes with potential were identified by the LASSO, as depicted in Figure 6A and B. Subsequently, the RF algorithm ranked the genes based on their respective importance levels (Figure 6C).^23^ By utilizing a Venn diagram, we observed an overlap between the top 10 most important genes identified by RF and the 8 potential candidate genes identified by LASSO. Consequently, six genes (CD3D, GZMA, IL2RB, KLRC1, CD5 and HLA‐DQA1) were selected for further validation. Additionally, it was noted that these six genes exhibit interactions with each other through intermediate molecules.

### 3.6 Diagnostic value assessment

**Figure 7:**
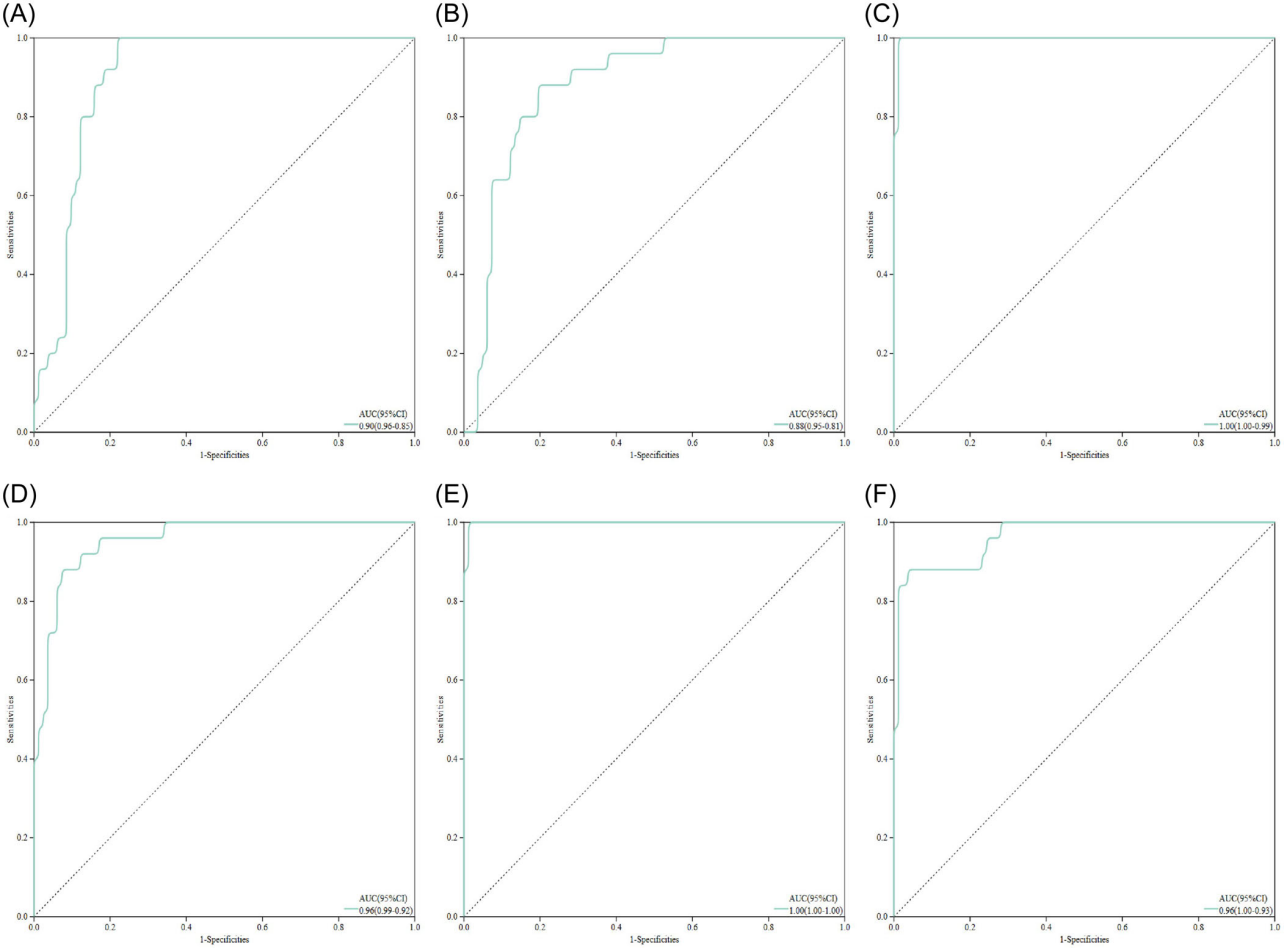
ROC curves for each candidate gene.

ROC curves were constructed based on six candidate central genes to assess diagnostic specificity and sensitivity for each gene. We calculated the AUC and 95% confidence interval for each project. The results are as follows: CD3D (AUC 0.90, CI 0.85 ‐−0.96), GZMA (AUC 0.88, CI 0.81 ‐−0.95), IL2RB (AUC 1.0, CI 0.99 ‐−1.00), KLRC1 (AUC 0.96, CI 0.92–0.99), CD5 (AUC 1.00, CI 1.00–1.00), and HLA‐DQA1 (AUC 0.96, CI 0.93–1.000) (Figure 7A‐F). The candidate genes exhibit high diagnostic potential for COVID‐19 in conjunction with sepsis.

### 3.7 Hub gene validation

**Figure 8:**
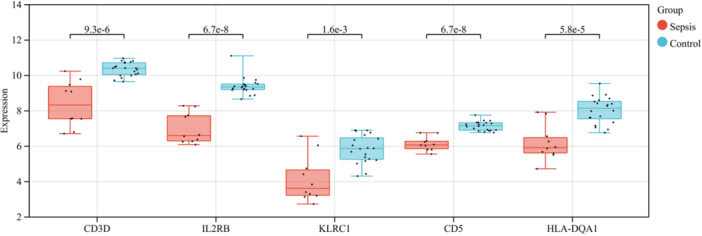
Hub Gene Validation. The boxplot analysis revealed that the expression levels of CD3D, IL2RB, KLRC1，CD5 and HLA‐DQA1 exhibited a significant decrease in GSE28750.

We utilized the external data set GSE28750 to further validate genes potentially associated with the concurrent presence of COVID‐19 and sepsis. Subsequently, we selected five genes (CD3D, IL2RB, KLRC1，CD5 and HLA‐DQA1) with an AUC exceeding 0.9 for subsequent validation. The expression levels of five hub genes were found to be lower in sepsis patients compared to healthy controls (Figure 8).

### 3.8 Immune infiltration analysis

In our study, we noted that the genes associated with COVID‐19 may be associated with the immune regulation of sepsis. Thus, immune cell infiltration analysis could better elucidate the relationship between the five key genes and immune cells.

**Figure 9:**
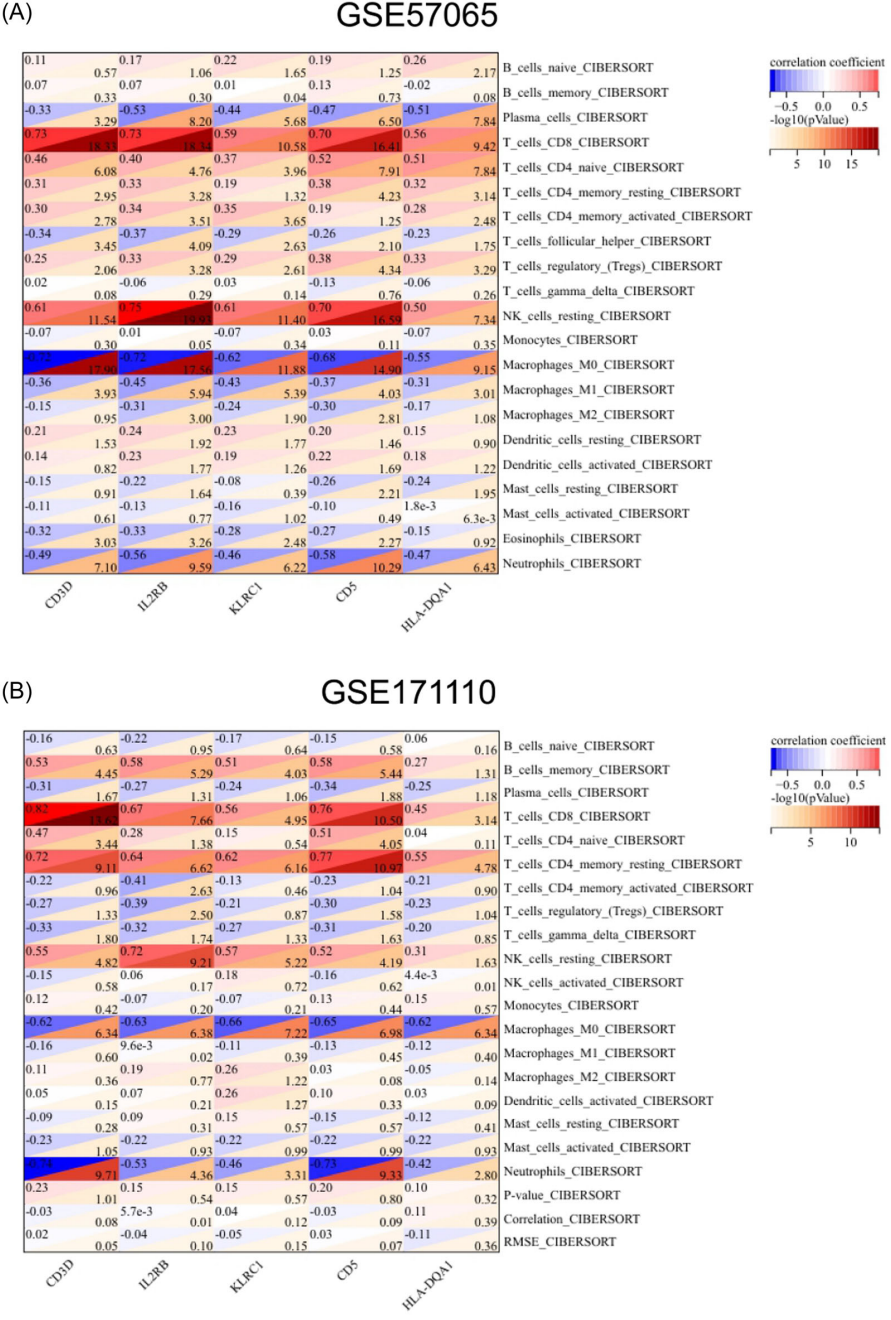
Association between the hub genes and immune infiltration. (A) In GSE57065, a positive correlation between the hub gene and the level of immune cell infiltration is observed in the red group, while a negative correlation is noted in the blue group. (B) Correlation results between the hub gene and GSE171110 immune cell infiltration are presented.

A detailed analysis of the relationship between five key genes and immune cells was conducted using the “Cibersort” R package and Spearman correlation analysis. As depicted in Figure 9A, the five hub genes were predominantly associated with CD8T cells, NK‐resting cells, and M0 macrophages in the sepsis sample GSE57065. Compared to healthy controls, in COVID‐19, the core gene CD3D demonstrated a positive correlation with CD8T cells, IL2RB exhibited a positive correlation with NK resting cells, KLRC1 exhibited a negative correlation with M0 macrophages, CD5 revealed a positive correlation with CD4T cells, and HLA‐DQA1 displayed a negative correlation with M0 macrophages.

## 4 DISCUSSION

Sepsis is a grave public health challenge and the primary cause of mortality in intensive care units. Recent investigations have unearthed several novel biomarkers for the diagnosis of sepsis, including Urokinase plasminogen activator receptor, Pro‐adrenomedullin, and CD64. However, few studies have investigated the interaction between sepsis and COVID‐19. In this research, we implemented an array of integrated bioinformatics analyses and machine‐learning techniques to assess the diagnostic potential of sepsis in COVID‐19 patients. Five key candidate genes (CD3D, IL2RB, KLRC1，CD5 and HLA‐DQA1) were validated using external datasets.

All data samples utilized in this study were derived from peripheral blood specimens. Hence, by specifically procuring peripheral blood samples from COVID‐19 patients and evaluating the expression of five immune‐related genes, we can effectively infer the probability of sepsis in these individuals. The utilization of peripheral blood assays for diagnosing various diseases has gained widespread acceptance, exemplifying a pragmatic and efficient clinical approach.

T lymphocytes execute a vital function in the adaptive immune response by acknowledging antigens derived from pathogens. CD3D encodes proteins involved in T‐cell development and signaling. The results of gene expression profiling studies conducted on 74 patients undergoing sepsis surgery revealed a negative correlation between CD3D and both Sepsis‐related Organ Failure scores and sepsis mortality.^24^ Deficiency of CD3D may contribute to immune damage.^25^ In a case‐control study examining 23 inflammation‐related genes, the combination of CD3D, TNF, and IL1B demonstrated notable specificity, yielding a negative predictive value of 98.1%.^26^

IL2RB is involved in T cell‐mediated immune response as a protein‐coding gene. RNA sequencing findings demonstrated a downregulation of IL2RB expression in sepsis, which was significantly associated with the prognosis of the condition.^24^, ^27^, ^28^ The cell transfection experiment demonstrated that IL2RB has the potential to enhance the balance between Th1 and Th2 responses in sepsis‐induced immune dysfunction.^28^ The inhibition of IL2RB has been demonstrated to mitigate acute lung injury in septic mice.^29^ Potential therapeutic targeting of IL2RB appears to offer a promising approach for sepsis treatment.

KLRC1, as a family of killer cell lectin‐like receptors, is a transmembrane protein predominantly expressed in NK cells, mediating lysis of certain tumor cells and virus‐infected cells. KLRC1 is primarily engaged in cell depletion and functions as an immunosuppressive checkpoint within the tumor microenvironment, potentially leading to the impairment of NK cell and tumor‐specific T cell functionality.^30^ To date, there is limited research on sepsis, necessitating further investigation to elucidate.

CD5 is extensively expressed on the surface of thymocytes, T lymphocytes, and B lymphocyte subsets, and it is implicated in the regulation of T cell activation, as well as modulating TCR signaling. The level of soluble CD5 lymphocyte surface receptor is positively correlated with the SOFA score, and a higher mortality rate is observed in ICU patients when the concentration of soluble CD5 lymphocyte surface receptor exceeds 1500 ng/ml.^31^ Another study found that a soluble form of the CD5 lymphocyte surface receptor not only binds but aggregates fungal cells, which may prevent pathogen transmission and facilitate pathogen clearance.^32^ In consequence, the soluble form of the CD5 lymphocyte surface receptor appears to hold greater relevance in fungal infection.^32^

HLA‐DQA1 performs a crucial function in the immune system by presenting extracellular protein peptides.^33^ In the analysis of multiple gene expression profiles, HLA‐DQA1 demonstrated a high predictive performance with an AUC > 0.95, and the HLA classifier emerged as an independent prognostic predictor. The results from qRT‐PCR also confirmed a lower expression level of HLA‐DQA1 in sepsis.^34^ Previous studies have demonstrated that low‐risk HLA subgroups exhibit relatively preserved immune function and reduced mortality, whereas high‐risk HLA subgroups exhibit impaired immune function and elevated mortality. The application of HLA classifier‐based treatment guidelines may potentially yield benefits for sepsis patients.^35^ As a member of the HLA class II, mHLA‐DQ has emerged as a robust biomarker for evaluating immune suppression, and it is extensively employed to orientate immunomodulatory treatment.^36^ HLA‐DQA1 can effectively respond to immunity and is expected to be a diagnostic target for sepsis in COVID‐19.

Our study has certain limitations. Firstly, although additional datasets were incorporated to validate the diagnostic value, there is a lack of subsequent experimental validation. Secondly, one limitation of this study is the lack of a specific data set of COVID‐19 patients who progressed to sepsis versus those who did not. As a result, the findings are based on the intersection of significantly expressed genes in independently analyzed sepsis and COVID‐19 datasets, which limits our ability to make direct conclusions about sepsis progression in COVID‐19 patients. Another limitation is that while we independently analyzed sepsis and COVID‐19 datasets to identify common immune dysregulation pathways, this approach does not directly predict COVID‐19 patients at risk of sepsis. Additionally, the validation cohort used was specific to sepsis and not COVID‐19, which may limit the applicability of the findings to COVID‐19‐related sepsis. Moreover, we did not explore whether the incidence of sepsis‐related deaths in COVID‐19 has plateaued or decreased over time. This is a crucial area of investigation that was outside the scope of this study but would provide valuable insight into the current state of sepsis in COVID‐19. Lastly, it is crucial to emphasize the significance of exploring causality and integrating machine learning for accurate biomarker prediction.^37^, ^38^

## 5 CONCLUSION

CD3D, CD5, HLA‐DQA1, IL2RB, and KLRC1 were diagnostic candidate genes for Covid‐19 patients with sepsis.

## AUTHOR CONTRIBUTIONS

**Jiuang Li**: Conceptualization; Data curation; Formal analysis; Investigation; Methodology; Project administration; Resources; Software; Supervision; Validation; Visualization; Writing—original draft; Writing—review and editing. **Shiqian Pu**: Investigation; Methodology; Visualization; Writing—original draft. **Lei Shu**: Data curation; Formal analysis; Methodology; Resources; Software; Supervision; Validation; Visualization; Writing—original draft. **Mingjun Guo**: Data curation; Formal analysis; Methodology; Resources; Software; Supervision; Validation; Visualization; Writing—original draft. **Zhihui He**: Conceptualization; Data curation; Formal analysis; Funding acquisition; Investigation; Methodology; Project administration; Resources; Supervision; Writing—original draft; Writing—review and editing.

## CONFLICT OF INTEREST STATEMENT

The authors declare no conflicts of interest.

## Data Availability

GEO belongs to public databases. The patients involved in the database have obtained ethical approval. Users can download relevant data for free for research and publish relevant articles. Our study is based on open‐source data, so there are no ethical issues and other conflicts of interest.

## References

[1] Fleischmann C , Scherag A , Adhikari NKJ , et al. Assessment of global incidence and mortality of hospital‐treated sepsis. current estimates and limitations. Am J Respir Crit Care Med. 2016;193(3):259‐272. 10.1164/rccm.201504-0781OC 26414292

[2] Dong E , Du H , Gardner L . An interactive web‐based dashboard to track COVID‐19 in real time. Lancet Infect Dis. 2020;20(5):533‐534. 10.1016/s1473-3099(20)30120-1 32087114 PMC7159018

[3] Chen T , Wu D , Chen H , et al. Clinical characteristics of 113 deceased patients with coronavirus disease 2019: retrospective study. BMJ. 2020;368:m1091. 10.1136/bmj.m1091 32217556 PMC7190011

[4] Chao JY , Derespina KR , Herold BC , et al. Clinical characteristics and outcomes of hospitalized and critically ill children and adolescents with coronavirus disease 2019 at a tertiary care medical center in New York city. J Pediatr. 2020;223:14‐19.e2. 10.1016/j.jpeds.2020.05.006 32407719 PMC7212947

[5] Zhou F , Yu T , Du R , et al. Clinical course and risk factors for mortality of adult inpatients with COVID‐19 in Wuhan, China: a retrospective cohort study. Lancet. 2020;395(10229):1054‐1062. 10.1016/s0140-6736(20)30566-3 32171076 PMC7270627

[6] Lu L , Liu LP , Gui R , et al. Discovering common pathogenetic processes between COVID‐19 and sepsis by bioinformatics and system biology approach. Front Immunol. 2022;13:975848. 10.3389/fimmu.2022.975848 36119022 PMC9471316

[7] López‐Collazo E , Avendaño‐Ortiz J , Martín‐Quirós A , Aguirre LA . Immune response and COVID‐19: A mirror image of sepsis. Int J Biol Sci. 2020;16(14):2479‐2489. 10.7150/ijbs.48400 32792851 PMC7415424

[8] Fang C , Ma Y . Peripheral blood genes crosstalk between COVID‐19 and sepsis. Int J Mol Sci. 2023;24(3):2591. 10.3390/ijms24032591 36768914 PMC9916586

[9] Liu J , Li S , Liu J , et al. Longitudinal characteristics of lymphocyte responses and cytokine profiles in the peripheral blood of SARS‐CoV‐2 infected patients. EBioMedicine. 2020;55:102763. 10.1016/j.ebiom.2020.102763 32361250 PMC7165294

[10] Shenoy S . Coronavirus (Covid‐19) sepsis: revisiting mitochondrial dysfunction in pathogenesis, aging, inflammation, and mortality. Inflamm Res. 2020;69(11):1077‐1085. 10.1007/s00011-020-01389-z 32767095 PMC7410962

[11] Alberca RW , Solis‐Castro RL , Solis‐Castro ME , et al. Platelet‐Based biomarkers for diagnosis and prognosis in COVID‐19 patients. Life . 2021;11(10). 10.3390/life11101005 PMC8538377 34685377

[12] Zhang A , Xing L , Zou J , Wu JC . Shifting machine learning for healthcare from development to deployment and from models to data. Nat Biomed Eng. 2022;6(12):1330‐1345. 10.1038/s41551-022-00898-y 35788685 PMC12063568

[13] Morimont L , Dechamps M , David C , et al. NETosis and nucleosome biomarkers in septic shock and critical COVID‐19 patients: an observational study. Biomolecules. 2022;12(8):1038. 10.3390/biom12081038 36008932 PMC9405965

[14] Tjendra Y , Al Mana AF , Espejo AP , et al. Predicting disease severity and outcome in COVID‐19 patients: A review of multiple biomarkers. Arch Pathol Lab Med. 2020;144(12):1465‐1474. 10.5858/arpa.2020-0471-SA 32818235

[15] Arturi F , Melegari G , Giansante A , Giuliani E , Bertellini E , Barbieri A . COVID‐19 biomarkers for critically ill patients: A compendium for the physician. Neurol Int. 2023;15(3):881‐895. 10.3390/neurolint15030056 37489362 PMC10366869

[16] Riva G , Nasillo V , Luppi M , Tagliafico E , Trenti T . Linking COVID‐19, monocyte activation and sepsis: MDW, a novel biomarker from cytometry. EBioMedicine. 2022;75:103754. 10.1016/j.ebiom.2021.103754 34922322 PMC8672420

[17] Bellinvia S , Edwards CJ , Schisano M , Banfi P , Fallico M , Murabito P . The unleashing of the immune system in COVID‐19 and sepsis: the calm before the storm? Inflamm Res. 2020;69(8):757‐763. 10.1007/s00011-020-01366-6 32468151 PMC8823100

[18] Zhang WY , Chen ZH , An XX , et al. Analysis and validation of diagnostic biomarkers and immune cell infiltration characteristics in pediatric sepsis by integrating bioinformatics and machine learning. World J Pediatr. 2023;19(11):1094‐1103. 10.1007/s12519-023-00717-7 37115484 PMC10533616

[19] Komorowski M , Green A , Tatham KC , Seymour C , Antcliffe D . Sepsis biomarkers and diagnostic tools with a focus on machine learning. EBioMedicine. 2022;86:104394. 10.1016/j.ebiom.2022.104394 36470834 PMC9783125

[20] Liu VX , Walkey AJ . Machine learning and sepsis: on the road to revolution. Crit Care Med. 2017;45(11):1946‐1947. 10.1097/ccm.0000000000002673 29028697 PMC5657564

[21] Fan Y , Han Q , Li J , et al. Revealing potential diagnostic gene biomarkers of septic shock based on machine learning analysis. BMC Infect Dis. 2022;22(1):65. 10.1186/s12879-022-07056-4 35045818 PMC8772133

[22] Li P , Li T , Zhang Z , et al. Bioinformatics and system biology approach to identify the influences among COVID‐19, ARDS and sepsis. Front Immunol. 2023;14:1152186. 10.3389/fimmu.2023.1152186 37261353 PMC10227520

[23] Gao Y , Zhang G , Jiang S , Liu Y‐X . Wekemo bioincloud: A user‐friendly platform for meta‐omics data analyses. iMeta. 2024;3(1):e175. 10.1002/imt2.175 38868508 PMC10989175

[24] Almansa R , Heredia‐Rodríguez M , Gomez‐Sanchez E , et al. Transcriptomic correlates of organ failure extent in sepsis. J Infect. 2015;70(5):445‐456. 10.1016/j.jinf.2014.12.010 25557485

[25] Deng Q , Luo Y , Chang C , Wu H , Ding Y , Xiao R . The emerging epigenetic role of CD8+T cells in autoimmune diseases: A systematic review. Front Immunol. 2019;10:856. 10.3389/fimmu.2019.00856 31057561 PMC6482221

[26] Hinrichs C , Kotsch K , Buchwald S , et al. Perioperative gene expression analysis for prediction of postoperative sepsis. Clin Chem. 2010;56(4):613‐622. 10.1373/clinchem.2009.133876 20133891

[27] Zhang Q , Hu Y , Wei P , et al. Identification of hub genes for adult patients with sepsis via RNA sequencing. Sci Rep. 2022;12(1):5128. 10.1038/s41598-022-09175-z 35332254 PMC8948204

[28] Zhou J , Zhang Y , Zhuang Q . IL2RB affects Th1/Th2 and Th17 responses of peripheral blood mononuclear cells from septic patients. Allergol Immunopathol. 2023;51(3):1‐7. 10.15586/aei.v51i3.757 37169553

[29] Liang G , Li J , Pu S , He Z . Screening of sepsis biomarkers based on bioinformatics data analysis. J Healthc Eng. 2022;2022:6788569. 10.1155/2022/6788569 36199375 PMC9529510

[30] André P , Denis C , Soulas C , et al. Anti‐NKG2A mAb is a checkpoint inhibitor that promotes anti‐tumor immunity by unleashing both T and NK cells. Cell. 2018;175(7):1731‐1743.e13. 10.1016/j.cell.2018.10.014 30503213 PMC6292840

[31] Aibar J , Martínez‐Florensa M , Castro P , et al. Pattern of soluble CD5 and CD6 lymphocyte receptors in critically ill patients with septic syndromes. J Crit Care. 2015;30(5):914‐919. 10.1016/j.jcrc.2015.04.120 26031813

[32] Vera J , Fenutría R , Cañadas O , et al. The CD5 ectodomain interacts with conserved fungal cell wall components and protects from zymosan‐induced septic shock‐like syndrome. Proc Natl Acad Sci. 2009;106(5):1506‐1511. 10.1073/pnas.0805846106 19141631 PMC2635803

[33] Koşaloğlu‐Yalçın Z , Lanka M , Frentzen A , et al. Predicting T cell recognition of MHC class I restricted neoepitopes. Oncoimmunology. 2018;7(11):e1492508. 10.1080/2162402x.2018.1492508 30377561 PMC6204999

[34] Chen Z , Chen R , Ou Y , et al. Construction of an HLA classifier for early diagnosis, prognosis, and recognition of immunosuppression in sepsis by multiple transcriptome datasets. Front Physiol. 2022;13:870657. 10.3389/fphys.2022.870657 35685286 PMC9171028

[35] Steinhagen F , Schmidt SV , Schewe JC , Peukert K , Klinman DM , Bode C . Immunotherapy in sepsis ‐ brake or accelerate? Pharmacol Ther. 2020;208:107476. 10.1016/j.pharmthera.2020.107476 31931100

[36] Monneret G , Lepape A , Voirin N , et al. Persisting low monocyte human leukocyte antigen‐DR expression predicts mortality in septic shock. Intensive Care Med. 2006;32(8):1175‐1183. 10.1007/s00134-006-0204-8 16741700

[37] Zhang Z , Jin P , Feng M , et al. Causal inference with marginal structural modeling for longitudinal data in laparoscopic surgery: A technical note. Laparosc Endosc Robot Surg. 2022;5(4):146‐152. 10.1016/j.lers.2022.10.002

[38] Lecca P . Machine learning for causal inference in biological networks: perspectives of this challenge. Front Bioinform. 2021;1:746712. 10.3389/fbinf.2021.746712 36303798 PMC9581010

